# Prognostic significance of TRAIL-R3 and CCR-2 expression in tumor epithelial cells of patients with early breast cancer

**DOI:** 10.1186/s12885-017-3259-8

**Published:** 2017-04-18

**Authors:** Vivian Labovsky, Leandro Marcelo Martinez, Kevin Mauro Davies, María de Luján Calcagno, Hernán García-Rivello, Alejandra Wernicke, Leonardo Feldman, Ayelén Matas, María Belén Giorello, Francisco Raúl Borzone, Hosoon Choi, Scott C. Howard, Norma Alejandra Chasseing

**Affiliations:** 1 Instituto de Biología y Medicina Experimental, Laboratorio de Inmunohematología (IBYME) – Consejo Nacional de Investigaciones Científicas y Técnicas (CONICET), Vuelta de Obligado 2490, CP 1428 Ciudad Autónoma de Buenos Aires, Argentina; 2 Departamento de Anatomía Patológica, Hospital Italiano, Juan Domingo Perón 4190, CP 1181 Ciudad Autónoma de Buenos Aires, Argentina; 3 Departamento de Bioestadística, Facultad de Farmacia y Bioquímica, Universidad de Buenos Aires, Junín 954, CP 1113 Ciudad Autónoma de Buenos Aires, Buenos Aires Argentina; 4 Departamento de Trasplante de Medula Ósea, Fundación Favaloro, Solis 443, C1078AAI Ciudad Autónoma de Buenos Aires, Argentina; 5 Central Texas Veterans Research Foundation, Temple, TX USA; 6 University of Tennsseee Health Sciences Center, Memphis, USA

**Keywords:** Early breast cancer, Spindle-shaped stromal cells, Tumor epithelial cells, TRAIL-R3, CCR-2

## Abstract

**Background:**

Tumor epithelial cells (TEpCs) and spindle-shaped stromal cells, not associated with the vasculature, of patients with early breast cancer express osteoprotegerin (OPG), tumor necrosis factor-related apoptosis-inducing ligand (TRAIL), receptor activator of nuclear factor kappa B ligand, stromal cell derived factor-1, interleukin-6, macrophage colony stimulating factor, chemokine (C-C motif) ligand-2 (CCL-2) and their receptors at significantly higher levels compared with non-neoplastic breast tissues. We evaluated the clinicopathological significance of these ligands and receptors in TEpC and spindle-shaped stromal cells, not associated with the vasculature, to determine their impact on prognosis of patients with early-stage breast cancer.

**Methods:**

We conducted immunohistochemical analyses of protein expression in primary tumors of patients with early breast cancer and analyzed their association with standard prognostic parameters and clinical outcomes, including local relapse, metastatic recurrence, disease-free survival (DFS), metastasis-free survival (MFS), and overall survival (OS).

**Results:**

Elevated levels of TRAIL-R3 and chemokine (C-C motif) receptor 2 (CCR-2) in TEpCs and OPG and CCL-2 in stromal cells were significantly associated with a higher risk of metastasis (*p* = 0.032, *p* = 0.003, *p* = 0.038, and *p* = 0.049; respectively). Moreover, high expression of TRAIL-R3 and CCR-2 in TEpCs was associated with shorter DFS, MFS, and OS. High TRAIL-R3 expression in TEpCs was an independent prognostic factor for DFS and OS, and high CCR-2 expression in these cells was an independent prognostic factor for MFS.

**Conclusions:**

High levels of TRAIL-R3 and CCR-2 expression in TEpCs identified patients with early breast cancer with poor outcomes.

## Background

Breast cancer is the most common cancer among women worldwide [1–4] and in Argentina affects more than 25,000 women and causes more than 5000 deaths each year (Bureau of Health Information Statistics and Nation, Department of Statistics and Health Information, Ministry of Health, Argentina, 2013). Distant metastasis is the main cause of death in these patients [5]. In high-income countries, breast cancer is usually diagnosed early, and treatment with curative intent and manageable toxicity is feasible. However, many women experience recurrence despite receiving optimal therapy, likely because the tumor microenvironment plays a key role in the development of resistance to treatment [6].

Breast cancer tissue comprises tumor epithelial cells (TEpCs) and stromal cells such as mesenchymal stem cells, tumor-associated fibroblasts, fibroblasts, endothelial cells, adipocytes, and immune cells. The interaction of malignant and non-malignant cells influences tumorigenesis, tumor growth, metastasis, and response to therapy [6–15]. Our group demonstrated that spindle-shaped stromal cells are not associated with the vasculature and TEpCs from primary invasive ductal breast cancer in women with stage I or II express molecules such as osteoprotegerin **(**OPG), tumor necrosis factor-related apoptosis-inducing ligand (TRAIL), receptor activator of nuclear factor kappa B ligand **(**RANKL), stromal cell derived factor **(**SDF)-1, interleukin (IL)-6, chemokine (C-C motif) ligand-2 (CCL-2) and their receptors [15]. These molecules, which are likely involved in the interactions between these cell types, mediate proliferation, survival, migration, and intravasation of TEpCs as well as angiogenesis in the primary tumor [15]. These findings led us to ask whether the levels of expression of these ligand-receptor pairs are useful for predicting the outcomes of patients with stage I/II breast cancer.

## Methods

### Patients

We conducted a retrospective study of 63 consecutive patients (aged 42–80 years) with a confirmed histological diagnosis of breast cancer who underwent initial surgery at the Hospital Italiano of Buenos Aires, Argentina. Patients were included if they were diagnosed with stage I/II invasive ductal breast cancer according to the International Union Against Cancer TNM classification system [16] and ≥10 years after surgery. Exclusion criteria included neoadjuvant therapies, lack of tissue, and another primary tumor. After surgery, all patients were treated with the indicated therapy, depending on their clinical status and the histopathological characteristics of their tumor, which were determined according to the recommendations of the European Society for Medical Oncology [17]. The Instituto de Biología y Medicina Experimental and the Hospital Italiano Ethics Committees approved this study, and informed consent was obtained from patients or the relatives of deceased patients, in accordance with the principles of the Helsinki Declaration. Physicians who were unaware of the pathology results acquired clinical information from patients’ medical records, and the anonymity of the data was ensured using a code made available only to the biostatistician.

### Tumor samples

Breast tissues embedded in paraffin blocks and fixed in 10% neutral-buffered formalin were retrieved from the surgical archives, and 4-μm thick sections were used in the experiments described below.

### Analysis of protein expression

These tissues were processed and immunohistochemistry was used to determine the levels of ligands and receptors in TEpC and in spindle-shaped stromal cells, not associated with the vasculature, and it was completed as described in a previously work [15].

Immunoreactivity was reviewed and scored independently by two pathologists who were blinded to patient outcomes. In uncertain cases, re-evaluation was performed using a double-headed microscope, and staining was evaluated until a consensus was achieved. The agreement in immunohistochemical evaluation between the two observers was 91.77% (Cohen’s kappa coefficient = 0.895). Each sample was assayed in duplicate and was initially examined at 100× magnification followed by observation of five representative fields at 400× magnification along a projected Z-line. Expression levels were evaluated separately for the TEpCs and spindle-shaped stromal cells, not associated to the vasculature, per the percentage of positive cells and staining intensity, which were estimated according to the Allred score [15, 16]. The percentages of positive cells were assigned scores as follows: 0 (<10%), 1 (10%–30%), 2 (31%–60%), 3 (61%–90%), and 4 (>90%). Staining intensity was scored as 0 (no staining), 1 (weak), 2 (moderate), and 3 (strong), according to the relative intensity of staining of TEpCs analyzed using the anti-cytokeratin antibody [15, 18]. The final staining score was calculated using the sum of the percentage of positive cells and the staining intensity score, which ranged from 0 to 7. Stromal cells included in this study had a spindle shape and were not associated with vasculature. CD34 expression was undetectable in this type of stromal cells as previously reported [18].

### Patients’ clinicopathological characteristics

Classical prognostic markers were categorized according to cut-offs used in the protocols of the Hospital Italiano, [17] including: *a)* age < 50 or ≥50 years; *b)* tumor size <2 or ≥2 cm; *c)* histological grade according to the Scarff-Bloom-Richardson grading system [19], which is expressed as differentiated (G1), intermediate (G2), and poor (G3); *d)* expression of estrogen/progesterone receptors and HER2/neu was defined as negative or positive according to Wernicke et al. [17]; and *e)* presence of regional metastatic lymph nodes was recorded as negative (negative nodes in axillary dissection or sentinel lymph node) or positive (including micrometastasis) (Table 1).

**Table 1:** Clinical characteristics of 63 patients with early invasive ductal breast cancer

Characteristics	Patients (n)	Patients (%)
Age (years)		
< 50	10	15.9
≥ 50	53	84.1
Unknown	-	-
Tumor size (cm)		
<2	45	71.4
≥ 2	17	27.0
Unknown	1	1.6
Histological grade		
G1	15	23.8
G2	22	34.9
G3	24	38.1
Unknown	2	3.2
HER2/neu status		
Negative	39	61.9
Positive	23	36.5
Unknown	1	1.6
ER status		
Negative	15	23.8
Positive	47	74.6
Unknown	1	1.6
PR status		
Negative	14	22.2
Positive	48	76.2
Unknown	1	1.6
Regional lymph nodes		
Negative	44	69.8
Positive	16	25.4
Unknown	3	4.8
Local relapse		
Negative	50	79.4
Positive	6	9.5
Unknown	7	11.1
Metastatic event		
Non-metastasis	45	71.4
Metastasis	11	17.5
Unknown	7	11.1

### Statistical analysis

To evaluate the statistical significance of the associations between the expression of ligand or receptor and patients’ clinicopathological characteristics, we determined an optimal cut-off value according to a previous study [18]. The cut-off value was used to assign protein expression in tumor samples as negative/low or high. To determine the optimal cut-off value, the first quartile (Q1), median, and third quartile (Q3) values were tested individually using univariate analysis and compared with OS. The cut-off value with lowest *p* value was chosen. The optimal cut-off values for protein expression in TEpCs were as follows: OPG = 6 (Q3), TRAIL =6 (Q3), TRAIL-R1 = 0 (Q1), TRAIL-R2 = 6 (Q3), TRAIL-R3 = 5 (median), TRAIL-R4 = 5 (Q1), RANKL =3 (Q1), RANK =6 (Q1), SDF-1 = 5 (Q1), CXCR-4 = 4 (Q1), IL-6 = 4 (median), IL-6R = 6 (median), CCL-2 = 6 (Q3), and chemokine (C-C motif) receptor 2 (CCR-2) = 6 (Q3). The optimal cut-off values for protein expression in spindle-shaped stromal cells, not associated with vasculature, were as follows: OPG = 2 (median), TRAIL =4 (median), RANKL =2 (Q1), SDF-1 = 2 (Q1), IL-6 = 4 (Q3), and CCL-2 = 3 (Q3). We used Fisher’s exact test to evaluate the association between the expression of these proteins with classical prognostic markers as well as local relapse and metastatic occurrence. Moreover, the association between the ligand and receptor expressions in TEpCs and spindle-shaped stromal cells and metastatic occurrence is displayed as a heat map prepared using Excel.

DFS and MFS were defined as the interval from date of surgery to the first observation of tumor occurrence (metastatic occurrence and/or local relapse) and metastatic occurrence, respectively, or last follow-up. The interval from the date of surgery until death or last follow-up was defined as OS. Univariate analyses of DFS, MFS, and OS were performed using the Kaplan-Meier method, and the differences were evaluated using the log-rank (Mantel-Cox) test. When significant variables were identified, we applied the Cox proportional hazards model to the multivariate survival analysis using backward stepwise selection (likelihood ratio) that incorporated only the significant variables. Statistical analysis was performed using SSPS software (version 18.00, Chicago, Illinois) and InfoStat (version 2012, InfoStat Group, National University of Cordoba, Argentina). A two-sided *p* value <0.05 was considered statistically significant.

## Results

### Association of expression in TEpCs of OPG, TRAIL, RANKL, SDF-1, IL-6, and CCL-2 with patients’ clinicopathological characteristics

The expression of TRAIL was significantly associated with lymph node status (Table 2). High TRAIL expression was detected in 10/40 breast cancer patients with negative lymph nodes, and TRAIL expression was undetectable in 0/16 of patients with positive lymph nodes. SDF-1 expression was significantly associated with tumor size and was high in 31/44 patients with tumors <2 cm and in 7/17 patients with tumors ≥2 cm (*p* = 0.004, Table 2). High levels of CCL-2 expression were detected in some patients with negative (3/15) or positive (1/42) ER expression (Table 2). The DFS of patients with high CCL-2 expression was 67.7 ± 32.0 months, compared with 123.15 ± 8.28 months for those with low/negative CCL-2 expression (*p* = 0.048, Table 3).

**Table 2:** Association of the levels of expression of OPG, TRAIL, RANKL, SDF-1, IL-6, and CCL-2 in TEpCs with the clinicopathological characteristics of patients with early invasive

Characteristics		Ligands in TEpC											
		OPG		TRAIL		RANKL		SDF-1		IL-6		CCL-2	
		High expression %	*p*	High expression %	*p*	High expression %	*p*	High expression %	*p*	High expression %	*p*	High expression %	*p*
Age (years)	<50	10.0	>0.999	2.2	0.657	70.0	>0.999	40.0	0.153	22.2	0.464	22.2	0.110
	≥50	15.7		16.0		73.1		67.3		39.2		4.1	
Tumor size (cm)	<2	13.9	>0.999	17.1	>0.999	65.9	0.114	70.4	0.044*	42.9	0.236	7.3	>0.999
	≥2	11.8		17.6		88.2		41.2		23.5		6.2	
Histological grade	G1	20.0	0.713	21.4	0.744	46.7	0.073	80.0	0.093	42.9	0.307	6.7	0.357
	G2	10.0		20.0		80.9		66.7		45.0		0.0	
	G3	12.5		13.0		79.2		45.8		25.0		13.0	
HER2/neu status	Negative	8.1	0.239	18.9	0.733	76.3	0.388	63.2	>0.999	39.5	0.780	2.9	0.287
	Positive	21.7		14.3		65.2		60.9		33.33		13.6	
ER status	Negative	13.3	>0.999	7.7	0.429	80.0	0.523	40.0	0.064	42.9	0.753	20.0	0.049*
	Positive	13.3		20.0		69.6		69.6		35.6		2.4	
PR status	Negative	14.3	>0.999	8.3	0.444	78.6	0.737	50.0	0.351	38.5	>0.999	7.1	>0.999
	Positive	13.0		19.6		70.2		66.0		37.0		7.0	
Regional lymph nodes	Negative	11.6	0.666	25.0	0.048*	67.4	0.352	58.1	0.555	39.0	0.761	4.7	0.349
	Positive	20.0		0.0		81.2		68.7		31.2		14.3	
Metastatic occurrence	Negative	20.9	0.180	21.4	0.664	70.4	0.707	59.1	>0.999	30.2	>0.999	2.4	0.109
	Positive	18.2		10.0		81.8		63.6		40.0		18.2	
Local relapse	Negative	6.1	0.330	20.8	0.576	70.0	0.307	60.0	>0.999	38.8	0.284	4.3	0.266
	Positive	20.0		0.0		100.0		60.0		0.0		20.0

**Table 3:** Univariate analysis of disease-free, metastasis-free, and overall survival of patients with early invasive ductal breast cancer

Univariate	*p*-value		
	Disease-free survival	Metastasis-free survival	Overall survival
Age	0.598	0.448	0.500
Tumor size	0.113	0.020*	0.069
Histological grade	0.178	0.291	0.207
HER2/neu status	0.536	0.293	0.103
ER status	0.336	0.191	0.175
PR status	0.691	0.946	0.521
Regional lymph nodes	0.595	0.805	0.620
OPG/TEpC	0.167	0.052	0.178
TRAIL/TEpC	0.465	0.648	0.304
RANKL/TEpC	0.156	0.267	0.307
SDF-1/TEpC	0.660	0.932	0.710
IL-6/TEpC CCL-2	0.873	0.710	0.487
CCL-2/TEpC	0.048*	0.071	0.507
TRAIL-R1/TEpC	0.536	0.339	0.626
TRAIL-R2/TEpC	0.894	0.749	0.392
TRAIL-R3/TEpC	0.009*	0.012*	0.015*
TRAIL-R4/TEpC	0.186	0.131	0.478
RANK/TEpC	0.546	0.991	0.804
CXCR-4/TEpC	0.164	0.255	0.175
IL-6R/TEpC	0.391	0.540	0.626
CCR-2/TEpC	0.013*	0.002*	0.049*
OPG/stromal cells	0.318	0.101	0.441
TRAIL/stromal cells	0.284	0.084	0.337
RANKL/stromal cells	0.139	0.052	0.222
SDF-1/stromal cells	0.792	0.734	0.306
IL-6/stromal cells CCL-2	0.218	0.093	0.168
CCL-2/stromal cells	0.104	0.076	0.505

### Association of expression in TEpCs of TRAIL-R1–4, RANK, CXCR-4, IL-6R, and CCR-2 with patients’ clinicopathological characteristics

**Fig. 1:**
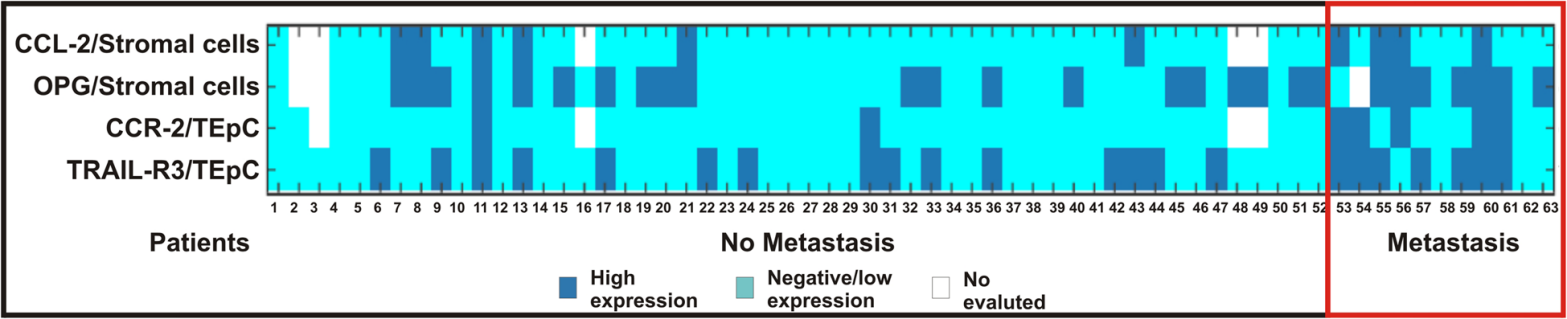
Heat map of the association of ligand and receptor expression in TEpCs and spindle-shaped stromal cells with metastasis. Graphic show data for tumor samples with high and negative/low expression of ligand and receptor

**Fig. 2:**
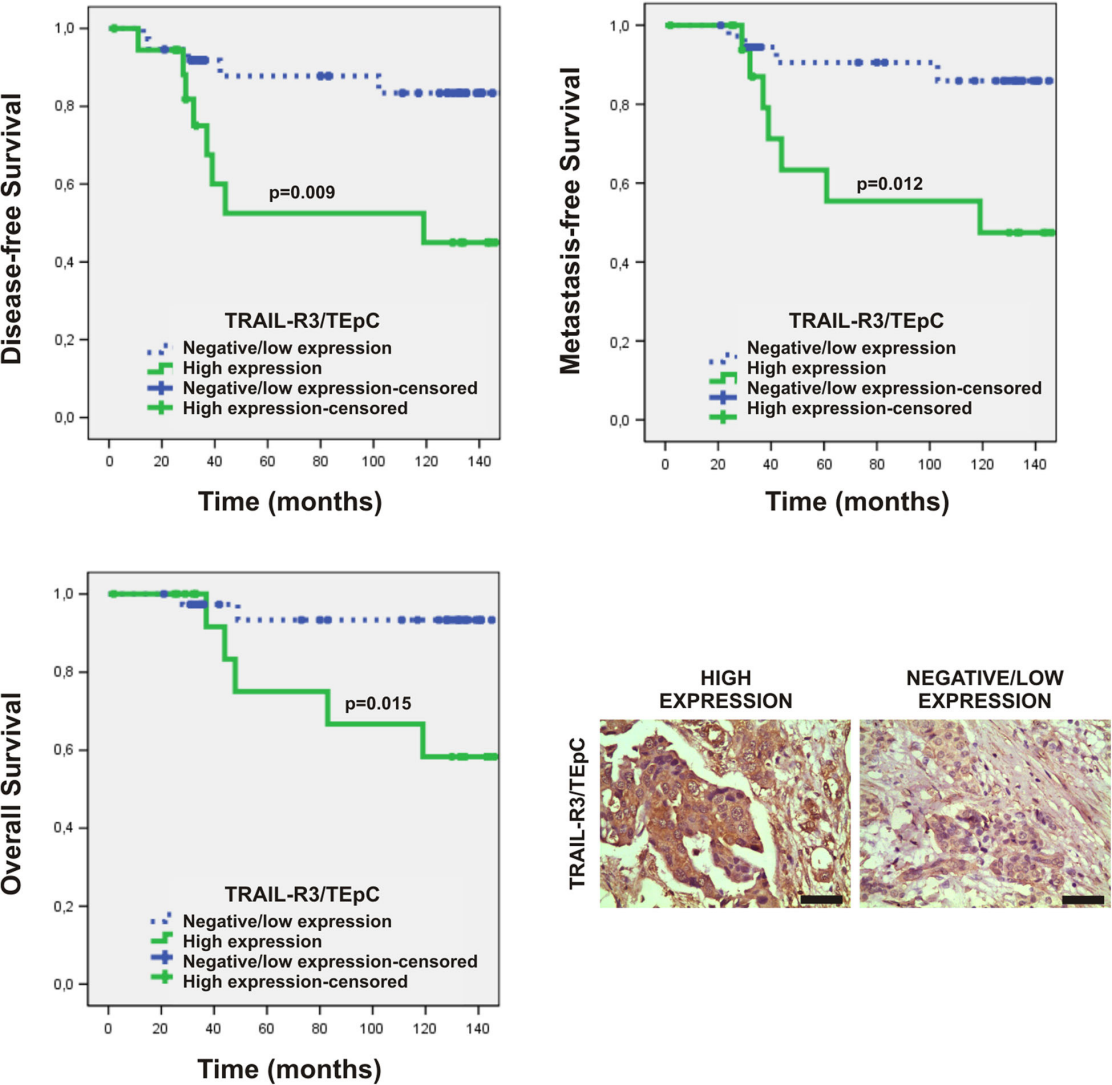
Association of TRAIL-R3 expression in TEpCs with DFS, MFS, and OS. Kaplan–Meier curves show representative data for tumor samples with high and negative/low expression of TRAIL-R3 in TEpCs. Original magnification: 400×. Scale bars =50 μm

IL-6R expression in TEpCs was associated with age (Table 4). Specifically, IL-6R expression was higher in 6/8 patients <50 years of age and in 15/48 patients ≥50 years of age (Table 4). Patients with high expression of TRAIL-R3 and CCR-2 in TEpCs were at significantly higher risk for metastatic tumors than patients with low expression (Table 4). High levels of TRAIL-R3 were expressed in 7/11 breast cancer patients with metastasis and in 12/45 patients with non-metastatic tumors (*p* = 0.032, Table 4). Certain patients with metastatic (5/11) or non-metastatic tumors (2/42) expressed high levels of CCR-2 (Fig. 1 and Table 4). There was an association of high TRAIL-R3 expression with shorter DFS, MFS, and OS (Table 3). The values of DFS, MFS and OS of patients with high TRAIL-R3 expression were as follows (months): 90.04 ± 14.64, 97.02 ± 14.08 and 112.75 ± 12.73, respectively; for patients with low/negative expression were 136.22 ± 7.52, 140.22 ± 6.61 and 146.51 ± 5.16, respectively (Fig. 2 and Table 3).

**Table 4:** Association of the levels of expression of TRAIL-R1-R4, RANK, CXCR-4, IL-6R, and CCR-2 in TEpCs with the clinicopathological characteristics of patients with early invasive ductal breast cancer

Characteristics		Receptors in TEpC															
		TRAIL-R1		TRAIL-R2		TRAIL-R3		TRAIL-R4		RANK		CXCR-4		IL-6-R		CCR-2	
		High expression %	*p*	High expression %	*p*	High expression %	*p*	High expression %	*p*	High expression %	*p*	High expression %	*p*	High expression %	*p*	High expression %	*p*
Age (years)	<50	87.5	0.249	0.0	>0.999	40.0	0.729	80.0	0.175	30.0	0.176	50.0	0.259	75.0	0.042*	11.1	>0.999
	≥50	63.5		9.6		34.0		54.7		55.8		73.1		31.3		12.0	
Tumor size (cm)	<2	66.7	>0.999	9.3	>0.999	35.6	>0.999	57.8	>0.999	50.0	0.579	68.2	>0.999	31.6	0.365	11.9	>0.999
	≥2	64.7		5.9		35.3		58.8		58.8		70.6		47.1		12.5	
Histological grade	G1	71.4	0.397	13.3	0.245	33.3	>0.999	60.0	0.803	53.3	>0.999	66.7	0.941	38.5	0.078	20.0	0.597
	G2	55.0		0.0		36.7		63.6		52.4		71.4		16.7		10.0	
	G3	75.0		12.5		37.5		54.2		50.0		66.7		50.0		8.7	
HER2/neu status	Negative	59.5	0.255	5.4	0.361	25.6	0.054	51.3	0.190	52.6	>0.999	71.0	0.776	26.5	0.083	16.7	0.235
	Positive	77.3		13.0		52.2		69.6		52.2		65.2		52.4		4.5	
ER status	Negative	66.7	>0.999	13.3	0.590	53.3	0.125	60.0	>0.999	60.0	0.562	73.3	0.757	46.7	0.361	20.0	0.359
	Positive	65.9		6.7		29.8		57.4		50.0		67.4		32.5		9.3	
PR status	Negative	64.3	>0.999	14.3	0.581	42.9	0.539	57.1	>0.999	57.1	0.766	78.6	0.516	35.7	>0.999	14.3	>0.999
	Positive	66.7		6.5		33.3		58.3		51.1		66.0		36.6		11.4	
Regional lymph nodes	Negative	64.3	0.342	9.3	>0.999	36.4	>0.999	61.4	0.771	55.8	0.558	72.1	0.533	35.0	0.749	11.9	>0.999
	Positive	80.0		6.7		37.5		56.2		43.7		62.5		42.9		13.3	
Metastatic occurrence	Negative	66.7	0.108	4.6	0.502	26.7	0.032*	51.1	0.092	50.0	>0.999	70.4	0.473	30.8	0.171	4.8	0.003*
	Positive	90.9		9.1		63.6		81.8		45.4		54.5		54.5		45.4	
Local relapse	Negative	70.8	>0.999	4.1	0.257	31.4	0.323	56.9	>0.999	48.0	0.669	70.0	0.316	35.6	>0.999	14.6	0.601
	Positive	80.0		20.0		60.0		60.0		60.0		40.0		40.0		0.0

**Fig. 3:**
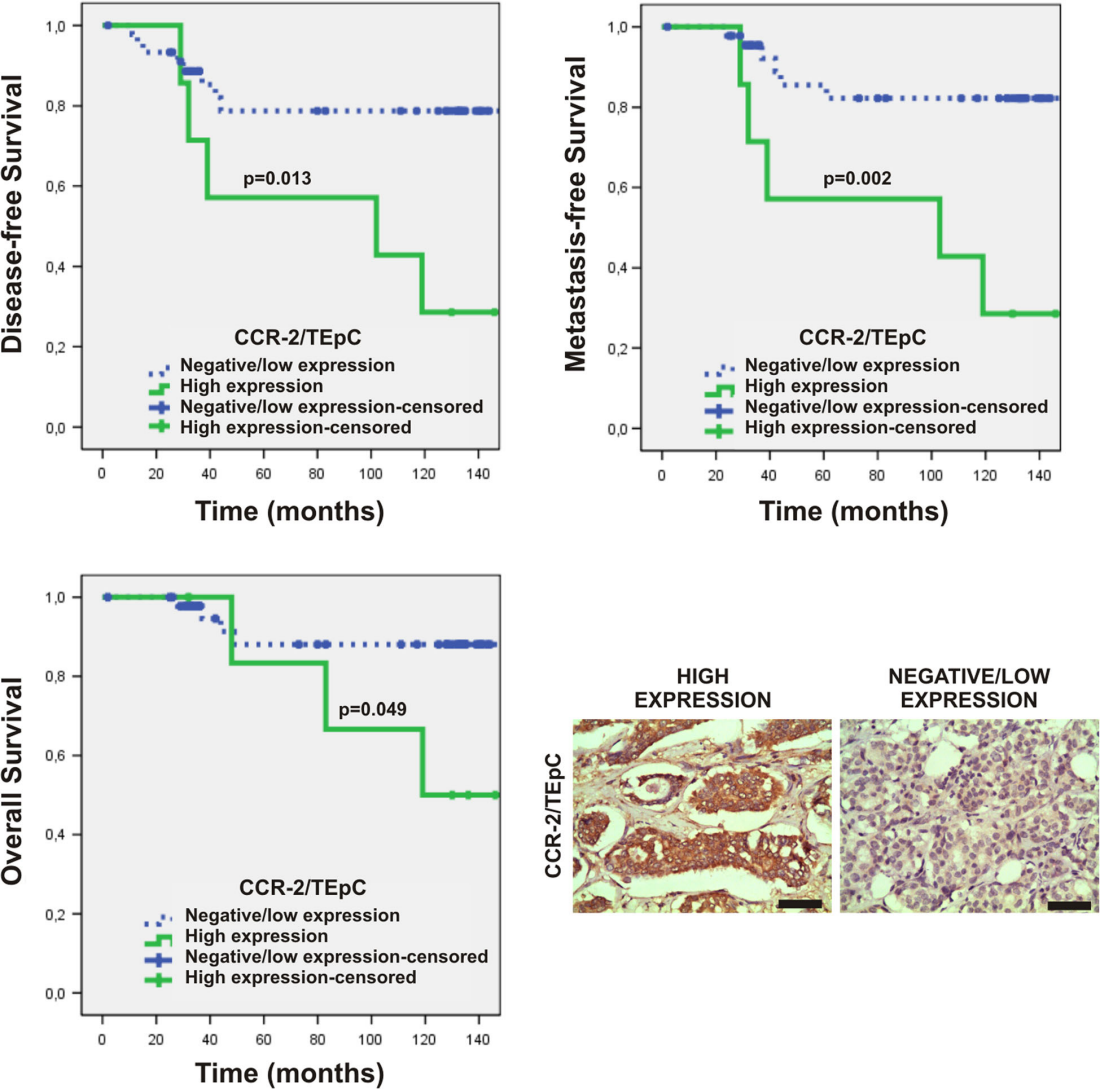
Association of CCR-2 expression in TEpCs with DFS, MFS, and OS. Images show representative data of tumor samples with high and negative/low expression of CCR-2 in TEpCs. Original magnification: 400×. Scale bars =50 μm

Furthermore, there was an association of high CCR-2 expression with shorter DFS, MFS and OS (Table 3). The values of DFS, MFS, and OS of patients with high CCR-2 expression were as follows (months): 87.57 ± 18.57, 87.71 ± 18.58, and 114.67 ± 15.29, respectively; for patients with low/negative expression were 127.57 ± 8.42, 133.94 ± 7.52, and 140.44 ± 6.41, respectively (Fig. 3 and Table 3).

### Association of expression in spindle-shaped stromal cells of OPG, TRAIL, RANKL, SDF-1, IL-6, and CCL-2 with patients’ clinicopathological characteristics

SDF-1 expression in spindle-shaped stromal cells was associated with histological grades, and high SDF-1 expression was detected in 10/15, 14/21, and 8/24 patients with differentiation grades G1, G2, and G3, respectively (Table 5). In contrast, high expression of OPG and CCL-2 in stromal cells was associated with a higher risk of metastasis (Fig. 1 and Table 5). High expression of OPG was observed in 7/10 patients with metastatic tumors and in 14/43 patients with non-metastatic tumors (*p* = 0.038, Fig. 1 and Table 5). In patients with metastatic or non-metastatic tumors, 4/11 and 4/44 expressed high levels of CCL-2, respectively (Fig. 1 and Table 5).

**Table 5:** Association of the levels of expression of OPG, TRAIL, RANKL, SDF-1, IL-6, and CCL-2 in spindle-shaped stroma cells (not associated with the vasculature) with the clinicopathological characteristics of patients with early invasive ductal breast cancer

Characteristics		Ligands in spindle-shaped stromal cells											
		OPG		TRAIL		RANKL		SDF-1		IL-6		CCL-2	
		High expression %	*p*	High expression %	*p*	High expression %	*p*	High expression %	*p*	High expression %	*p*	High expression %	*p*
Age (years)	<50	50.0	0.742	66.7	0.137	70.0	0.709	40.0	0.493	22.2	0.664	22.2	0.646
	≥50	44.0		35.3		75.0		55.8		17.6		16.3	
Tumor size (cm)	<2	48.8	0.255	38.1	>0.999	68.2	0.193	56.8	0.391	21.4	0.483	19.5	0.708
	≥2	31.2		41.2		88.2		41.2		11.8		12.5	
Histological grade	G1	60.0	0.336	35.7	0.518	60.0	0.491	66.7	0.042*	21.4	0.741	33.3	0.116
	G2	35.0		50.0		76.2		66.7		20.0		5.3	
	G3	39.1		33.3		79.2		33.3		12.5		17.4	
HER2/neu status	Negative	44.4	>0.999	44.7	0.272	78.9	0.368	57.9	0.302	18.4	>0.999	17.1	>0.999
	Positive	43.5		28.6		65.2		43.5		19.0		18.2	
ER status	Negative	46.7	>0.999	42.9	0.761	80.0	0.738	53.3	>0.999	21.4	>0.999	20.0	>0.999
	Positive	43.2		37.8		71.7		52.2		17.8		16.7	
PR status	Negative	42.9	>0.999	30.8	0.540	78.6	0.742	50.0	>0.999	15.4	>0.999	7.1	0.422
	Positive	44.4		41.3		72.3		53.2		19.6		20.9	
Regional lymph nodes	Negative	46.1	0.547	41.5	>0.999	74.4	0.745	55.8	0.773	17.1	>0.999	19.0	0.724
	Positive	35.7		37.5		68.7		50.0		18.7		14.3	
Metastatic occurrence	Negative	32.5	0.038*	34.9	0.072	65.9	0.146	47.3	0.745	11.6	0.163	9.1	0.049*
	Positive	70.0		70.0		90.9		54.5		30.0		36.4	
Local relapse	Negative	39.6	>0.999	42.9	0.633	72.0	0.621	50.0	>0.999	16.3	0.612	14.9	>0.999
	Positive	40.0		25.0		60.0		40.0		0.0		20.0	

### Univariate analysis of the association of classical prognostic markers with DFS, MFS, and OS

Of clinical variables analyzed, only tumor size was associated with MFS (Table 3). Patients with tumors >2 cm had earlier metastasis compared with those with tumors ≤2 cm as follows (months): 93.00 ± 15.59 vs 139.02 ± 6.47, respectively.

### Multivariate analysis

TRAIL-R3 expression in TEpCs was an independent prognostic factor for DFS and OS (Table 6). Moreover, tumor size and CCR-2 expression were independent prognostic factors for MFS (Table 6).

**Table 6:** Multivariate analysis of DFS, MFS, and OS of patients with early invasive ductal breast cancer

	Variables	HR	95% CI	*p*
Disease-free survival	TRAIL-R3 in TEpC	3.566	1.164–10.920	0.026
Metastasis-free survival	Tumor size	8.210	2.013–33.477	0.003
	CCR-2 in TEpC	10.257	2.569–40.947	0.001
Overall survival	TRAIL-R3 in TEpC	5.741	1.113–29.621	0.037

*C.I.* confidence interval, *HR* hazard ratio

## Discussion

Tumor progression is a multistep process involving interactions between tumor cells and spindle-shaped stromal cells, not associated with the vasculature, which supply signals that may promote tumor progression [15].

Here we show that high TRAIL expression in TEpCs was significantly associated with negative lymph-node status. Paracrine signaling induced by the binding of TRAIL to the death receptors TRAIL-R1 and TRAIL-R2 induces apoptosis [20–22]. Thus, the association of TRAIL expression in TEpCs of patients with negative lymph nodes might reflect the apoptotic effects of TRAIL that delay tumor progression as well as the extravasation of tumor cells to regional lymph nodes [23].

Patients with TEpCs that expressed high levels of TRAIL-R3 harbored metastases and experienced shorter DFS, MFS, and OS. TRAIL-R3 competes with TRAIL-R1, TRAIL-R2, or both for the binding of TRAIL, which inhibits apoptotic signaling [20]. Moreover, the expression of TRAIL-R3 in TEpCs was an independent prognostic marker for DFS and OS. These findings indicate the importance of evaluating TRAIL-R3 expression in TEpCs, because TRAIL is used to treat tumors. Thus, outcomes may be adversely affected by the level of TRAIL-R3 activity in tumors as well as in the tumor microenvironment.

In contrast, we found that high SDF-1 expression in TEpCs was significantly associated with tumor size <2 cm, which is consistent with the findings of previous studies [24, 25]. Furthermore, high expression of SDF-1 in spindle-shaped stromal cells, not associated with the vasculature, was significantly associated with conventional prognostic markers of less adverse tumor phenotypes, such as low histological grade (G1 and G2).

We show here that the expression of CCL-2 in TEpCs was associated with negative ER-status, which agrees with reports demonstrating that CCL-2 is overexpressed in ER-negative compared with ER-positive tumors [26]. These data suggest the involvement of CCL-2 in the progression of ER-negative breast tumors. Moreover high CCL-2 expression in TEpCs was significantly associated with DFS. CCL-2 directly promotes the malignant phenotype (epithelial mesenchymal transition) of TEpCs and increases their ability to migrate, proliferate, and invade tissues [27–30]. Also, patients with high CCR-2 expression in TEpCs experienced shorter DFS, MFS, and OS. Furthermore, the expression of CCR-2 is up-regulated in breast tumor cells, and knockdown of CCR-2 expression inhibits breast tumor development [31]. Additionally, we show here that CCR-2 expression was an independent prognostic factor for MFS.

Stromal cells such as fibroblast that produce CCL-2 enhance the invasiveness and metastatic growth of human breast cancer cell lines [31], which is consistent with the present findings of a significant association between high CCL-2 expressions in spindle-shaped stromal cells in patients with metastatic early-stage breast cancer. Our data indicate the importance of evaluating CCR-2 expression in TEpCs as well as CCL-2 expression in TEpC and spindle-shaped stromal cells, because the pathways that produce CCR-2 and its ligands may provide targets for the prevention of breast cancer progression and metastasis [29]. Interestingly, we found previously that the expression of CCL-2 in spindle-shaped stromal cells, not associated with the vasculature, correlated positively with the expression of CCR-2 in TEpCs, suggesting that CCL-2 signaling through CCR-2 may contribute to the interactions between TEpCs and spindle-shaped stromal cells, which enhance the malignant phenotype of tumor cells during the early stages of disease [15].

We uncovered a significant association between high OPG expression in spindle-shaped stromal cells and the presence of metastatic breast tumors. This finding is consistent with those showing that OPG produced by a breast tumor induces angiogenesis and inhibits TRAIL-mediated apoptosis to promote the growth of the primary tumor as well as metastatic cells [32, 33].

To our knowledge, this study is the first to demonstrate that high expression of TRAIL-R3 and CCR-2 in TEpCs serves as a prognostic marker of metastatic tumors as well as DFS, MFS, and OS in women with stage I/II invasive breast cancer. These new findings provide a rationale for further studies designed to target TRAIL-R3 and CCR-2 signaling pathways to facilitate the diagnosis, prevention, and treatment of breast cancer.

## Conclusions

High levels of TRAIL-R3 and CCR-2 expression in TEpCs identified early breast cancer patients with poor outcomes, including a higher risk of metastasis and shorter DFS, MFS, and OS and represent new independent prognostic factors that may also be suitable therapeutic targets.

## Abbreviations

DFS: Disease-free survival
MFS: Metastasis-free survival
OS: Overall survival
TEpCs: Tumor epithelial cells

## References

[1] Parkin DM, Bray F, Ferlay J, Pisani P (2001). Estimating the world cancer burden: Globocan 2000. Int J Cancer.

[2] Bhatia P, Sanders MM, Hansen MF (2005). Expression of receptor activator of nuclear factor-kappaB is inversely correlated with metastatic phenotype in breast carcinoma. Clin Cancer Res.

[3] Kakarala M, Wicha MS (2008). Implications of the cancer stem-cell hypothesis for breast cancer prevention and therapy. J Clin Oncol.

[4] Patel SA, Heinrich AC, Reddy BY, Srinivas B, Heidaran N, Rameshwar P (2008). Breast cancer biology: the multifaceted roles of mesenchymal stem cells. J Oncol.

[5] Rosa Mendoza ES, Moreno E, Caguioa PB (2013). Predictors of early distant metastasis in women with breast cancer. J Cancer Res Clin Oncol.

[6] Nantajit D, Lin D, Li JJ (2015). The network of epithelial-mesenchymal transition: potential new targets for tumor resistance. J Cancer Res Clin Oncol.

[7] Beacham DA, Cukierman E (2005). Stromagenesis: the changing face of fibroblastic microenvironments during tumor progression. Semin Cancer Biol.

[8] Wels J, Kaplan RN, Rafii S, Lyden D (2008). Migratory neighbors and distant invaders: tumor-associated niche cells. Genes Dev.

[9] Reddy BY, Lim PK, Silverio K, Patel SA, Won BW, Rameshwar P (2012). The microenvironmental effect in the progression, metastasis, and dormancy of breast cancer: a model system within bone marrow. Int J Breast Cancer..

[10] Rhodes LV, Antoon JW, Muir SE, Elliott S, Beckman BS, Burow ME (2010). Effects of human mesenchymal stem cells on ER-positive human breast carcinoma cells mediated through ER-SDF-1/CXCR4 crosstalk. Mol Cancer.

[11] Senst C, Nazari-Shafti T, Kruger S, Höner Zu Bentrup K, Dupin CL (2013). Prospective dual role of mesenchymal stem cells in breast tumor microenvironment. Breast Cancer Res Treat.

[12] Peng Q, Zhao L, Hou Y, Sun Y, Wang L, Luo H (2013). Biological characteristics and genetic heterogeneity between carcinoma-associated fibroblasts and their paired normal fibroblasts in human breast cancer. PLoS One.

[13] Khamis ZI, Sahab ZJ, Sang QX (2012). Active roles of tumor stroma in breast cancer metastasis. Int J Breast Cancer.

[14] Barcellos-de-Souza P, Gori V, Bambi F, Chiarugi P (2013). Tumor microenvironment: bone marrow-mesenchymal stem cells as key players. Biochim Biophys Acta.

[15] Labovsky V, Martinez LM, Davies KM, García-Rivello H, Calcagno Mde L, Matas A (2015). Association between Ligands and receptors related to the progression of early breast cancer in tumor epithelial and Stromal cells. Clin Breast Cancer.

[16] Edge S, Byrd DR, Compton CC, Fritz AG, Greene FL, Trotti A (2010). AJCC cancer staging manual and handbook, seventh edition, American joint committee on cancer.

[17] Wernicke M, Roitman P, Manfre D, Stern R. Breast cancer and the stromal factor. The "prometastatic healing process" hypothesis. Medicina (B Aires). 2011;71(1):15-21. 21296715

[18] Martinez LM, Labovsky V, Calcagno ML, Davies KM, Garcia Rivello H, Bianchi MS (2015). CD105 expression on CD34-negative spindle-shaped stromal cells of primary tumor is an unfavorable prognostic marker in early breast cancer patients. PLoS One.

[19] Bloom HJ, Richardson WW (1957). Histological grading and prognosis in breast cancer; a study of 1409 cases of which 359 have been followed for 15 years. Br J Cancer.

[20] Labovsky V, Vallone VB, Martinez LM, Otaegui J, Chasseing NA (2012). Expression of osteoprotegerin, receptor activator of nuclear factor kappa-B ligand, tumor necrosis factor-related apoptosis-inducing ligand, stromal cell-derived factor-1 and their receptors in epithelial metastatic breast cancer cell lines. Cancer Cell Int.

[21] Herbst RS, Eckhardt SG, Kurzrock R, Ebbinghaus S, O'Dwyer PJ, Gordon MS (2010). Phase I dose-escalation study of recombinant human Apo2L/TRAIL, a dual proapoptotic receptor agonist, in patients with advanced cancer. J Clin Oncol.

[22] Garimella SV, Gehlhaus K, Dine JL (2014). Identification of novel molecular regulators of tumor necrosis factor-related apoptosis-inducing ligand (TRAIL)-induced apoptosis in breast cancer cells by RNAi screening. Breast Cancer Res.

[23] Cross SS, Harrison RF, Balasubramanian SP, Lippitt JM, Evans CA, Reed MW (2006). Expression of receptor activator of nuclear factor kappabeta ligand (RANKL) and tumour necrosis factor related, apoptosis inducing ligand (TRAIL) in breast cancer, and their relations with osteoprotegerin, oestrogen receptor, and clinicopathological variables. J Clin Pathol.

[24] Lv ZD, Kong B, Liu XP, Dong Q, Niu HT, Wang YH (2014). CXCL12 chemokine expression suppresses human breast cancer growth and metastasis in vitro and in vivo. Int J Clin Exp Pathol.

[25] Mirisola V, Zuccarino A, Bachmeier BE, Sormani MP, Falter J, Nerlich A (2009). CXCL12/SDF1 expression by breast cancers is an independent prognostic marker of disease-free and overall survival. Eur J Cancer.

[26] Chavey C, Bibeau F, Gourgou-Bourgade S, Burlinchon S, Boissière F, Laune D (2007). Oestrogen receptor negative breast cancers exhibit high cytokine content. Breast Cancer Res.

[27] Soria G, Ben-Baruch A (2008). The inflammatory chemokines CCL2 and CCL5 in breast cancer. Cancer Lett.

[28] Potter SM, Dwyer RM, Hartmann MC, Khan S, Boyle MP, Curran CE (2012). Influence of stromal-epithelial interactions on breast cancer in vitro and in vivo. Breast Cancer Res Treat.

[29] Lu X, Kang Y (2009). Chemokine (C-C motif) ligand 2 engages CCR2+ stromal cells of monocytic origin to promote breast cancer metastasis to lung and bone. J Biol Chem.

[30] Klopp AH, Spaeth EL, Dembinski JL, Woodward WA, Munshi A, Meyn RE (2007). Tumor irradiation increases the recruitment of circulating mesenchymal stem cells into the tumor microenvironment. Cancer Res.

[31] Fang WB, Jokar I, Zou A, Lambert D, Dendukuri P, Cheng N (2012). CCL2/CCR2 chemokine signaling coordinates survival and motility of breast cancer cells through Smad3 protein- and p42/44 mitogen-activated protein kinase (MAPK)-dependent mechanisms. J Biol Chem.

[32] Weichhaus M, Chung ST, Connelly L (2015). Osteoprotegerin in breast cancer: beyond bone remodeling. Mol Cancer.

[33] Weichhaus M, Segaran P, Renaud A, Geerts D, Connelly L (2014). Osteoprotegerin expression in triple-negative breast cancer cells promotes metastasis. Cancer Med.

